# Enhancing point-of-care testing through standardized training and redeployment of pharmacy technicians in the community setting^☆^

**DOI:** 10.1016/j.rcsop.2021.100034

**Published:** 2021-06-10

**Authors:** Sarah Pope, Hunter Hill, Lindsey Cardosi, Lindsey Henson, Mike Wasson, Sara Stallworth, Kim Ward, Shane P. Desselle, Kenneth C. Hohmeier

**Affiliations:** a Kroger Health, United States of America; b Touro University California, College of Pharmacy, United States of America; c University of Tennessee Health Science Center, College of Pharmacy, United States of America

**Keywords:** Point-of-care testing (POCT), Community pharmacy, Pharmacy technician, Pharmacist, Pharmacy clinical services, Standardization of training, Workflow

## Abstract

**Objectives:**

The primary objective of this study was to assess the impact of a standardized training model for technician-supported point-of-care testing (POCT) on the number of health screenings performed across two states in a large community chain pharmacy. Secondary objectives included the assessment of pharmacist and technician perceptions of advanced roles of the pharmacy technician in POCT service delivery.

**Practice description:**

Certified pharmacy technicians (CPhTs) across six regional divisions of a large community chain pharmacy in Tennessee and Ohio participated in a standardized training program prior to implementation of technician-supported POCT.

**Practice innovation:**

Standardized training consisted of pre-training assessments, online training modules, post-training assessments, followed by in-person skills-based assessments. CPhT participation was limited to technical tasks of POCT (e.g. sample collections, quality assurance).

**Evaluation methods:**

The study addressed its primary objective by comparing total number of health screenings for included pharmacies in 2019 as compared to 2020. Descriptive and inferential statistics were used. Perceptions were assessed using an electronic, Likert-type scale questionnaire.

**Results:**

Pharmacies with technician-supported POCT showed a 46% increase in the total number of health screenings performed vs. 2019. The survey found that 74% (106/144) of pharmacists and 83% (34/41) of CPhTs agreed or strongly agreed that technician-supported POCT is acceptable for their practice site. Most pharmacy personnel agreed or strongly agreed that the service was appropriate and feasible for their respective practice sites.

**Conclusion:**

This study provided supporting evidence that technician-supported POCT may positively impact the number of health screenings conducted in a community pharmacy setting. Standardization of training may allow for expansion of this service across additional states. Furthermore, pharmacy personnel perceptions were overall positive.

## 1 Key points

What was already known:

• Point-of-care testing (POCT) is an increasingly popular service offered by community pharmacies, useful in areas such as diabetes, anticoagulation, and infectious disease.
• There is an opportunity to leverage the support of pharmacy technicians, allowing pharmacists to engage in high-quality patient care services.
• Standardized training requirements are imperative for pharmacy technicians to be effective in advanced roles.

What this study adds:

• Evidence that technician-supported POCT may positively impact the number of health screenings conducted in a community pharmacy setting.
• Standardization of training may allow for expansion of such a service across additional states.
• Overall, both pharmacist and technician perceptions of technician-supported POCT were positive.

## 2 Background

As community pharmacies adapt to the national shift towards a value-based healthcare model, pharmacists are evolving as key players in the delivery of direct patient care services.^1^, ^2^, ^3^ Pharmacists are widely recognized as the most accessible healthcare providers within communities placing them in a prime position to take a more active role in clinical decision making and providing disease management services such as medication therapy management and point of care testing (POCT).^4^ However, despite this unique opportunity to deliver highly accessible patient care, there has been a significant delay in the adoption of direct patient care services within community pharmacies over the last few decades.^2^^,^^5^^,^^6^ According to the 2019 national pharmacist workforce study, only about 43.9% of community pharmacists reported participation in comprehensive medication management services while a mere 19.6% of respondents provided point-of-care testing delivery within their pharmacies.^7^ This delayed uptake in the provision of pharmacist-delivered direct patient services can be attributed to existing barriers within community pharmacy workflow such as limited time to effectively deliver services, inadequate support staffing, lack of adequate training for pharmacy personnel, and a lack of reimbursement for delivery of these services.^1^^,^^2^^,^^5^^,^^6^^,^^8^

In an effort to overcome time and staffing constraints associated with delivery of direct patient care services, pharmacy technicians have been stepping into advanced clinical roles to fill existing gaps within the community pharmacy workflow process.^1^^,^^3^^,^^9^^,^^10^, ^11^, ^12^, ^13^ A review of current literature demonstrates that pharmacy technicians have been successful in adopting a variety of advanced responsibilities within the pharmacy such as immunization administration, tech-check-tech product verification, medication therapy management, medication reconciliation, and point of care testing delivery.^1^^,^^3^^,^^9^, ^10^, ^11^^,^^13^, ^14^, ^15^Evidence supporting the benefits of expanding technician roles from basic medication filling practices and administrative tasks to include direct patient care tasks is well established.^3^^,^^10^, ^11^, ^12^, ^13^^,^^16^ Based on their systematic review of 33 articles, Mattingly and colleagues concluded that organizations may be widely underutilizing pharmacy technicians in the delivery of direct patient care tasks.^16^ Furthermore, multiple studies demonstrate that the involvement of trained pharmacy technicians within advanced clinical roles can alleviate dispensing process time constraints and amplify opportunities for pharmacists to participate in clinical decision making and the provision of direct patient care.^2^^,^^3^^,^^10^^,^^13^^,^^16^ Allowing pharmacists to focus more directly on patient care services may provide a more cost-effective way to deliver value-based care within community pharmacies and lead to improved patient outcomes.^1^^,^^16^^,^^17^

POCT delivery within community pharmacies offers patients a unique opportunity to receive real-time, high quality lab testing and direct patient care during a single pharmacy encounter.^13^ POCT services can provide pharmacists and patients with the tools to conveniently manage diabetes, anticoagulation, cholesterol, influenza, and streptococcus-associated pharyngitis in the community setting, which can improve patient access to health care services and optimize patient health outcomes.^5^^,^^6^^,^^9^^,^^13^^,^^17^ Keller et al. concluded that the delivery of POCT services by trained advanced technicians may aid in increasing the efficiency of community pharmacy workflow, while Hill et al. demonstrated that pharmacy technician-supported POCT delivery can be successfully implemented into community pharmacy workflow and may positively influence health screening productivity within community pharmacies.^9^^,^^13^While the benefits of utilizing pharmacy extenders to assist in the delivery of clinical services within community pharmacy is well established and has been linked with improved technician job-related satisfaction, policies and standardized training programs for advanced clinical pharmacy technician roles such as POCT delivery are lacking.^16^^,^^18^, 19., 20

## 3 Objectives

The primary objective of this study was to assess the impact of a standardized training model for technician-supported POCT on the number of health screenings performed across two states in a large community pharmacy chain. The secondary objective of the study examined pharmacists' and pharmacy technicians' perceptions of technician engagement in the delivery of POCT services.

## 4 Methods

**Fig. 1:**
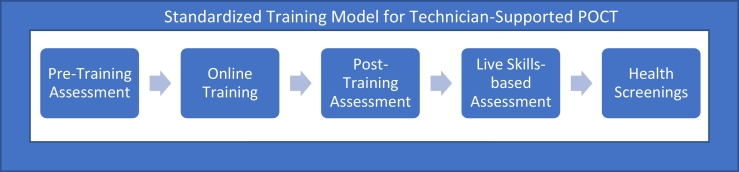
Description of the timeline of pharmacy technician-supported POCT. Pharmacy technicians were provided a pre-training assessment which provided a knowledge baseline of disease states, analyzer usage, and clinical empathy skills. Afterward, they completed a one-hour online training program at the pharmacy. This was followed by a live skills-based assessment. Upon passing the skills assessment, pharmacy technicians were qualified to provide POCT health screenings.

In this prospective quasi-experimental pre/post study, certified pharmacy technicians (CPhTs) across six regional divisions and two U.S. states of a large community chain pharmacy participated in a standardized training program for technician-driven POCT in preparation for an annual one-month free screening event available across 36 states of the large community chain pharmacy. The health screening included a combination of manual and automatic blood pressure measurements, as well as finger-stick blood sample collections to measure blood glucose and blood cholesterol. This model consisted of both online and live training (Fig. 1). The online modules were developed in collaboration with pharmacy leadership, University faculty, and an instructional design team. Approval for this project was granted by the UTHSC Institutional Review Board. Content focused on basic disease state education (e.g. diabetes, hypercholesterolemia), training on how to use the analyzer, and contained additional content focused on clinical empathy skills. Over 350 CPhTs completed the online training in Tennessee and Ohio. At least one technician was required to complete training from each participating location.

CPhTs were limited to the technical tasks of POCT including check-in paperwork, sample collection, and quality assurance tasks. Prior to beginning a health screening, technicians ensured that patients completed appropriate paperwork including a consent and statement of financial responsibility form, patient demographics, and a pre-screening questionnaire. Pharmacists completed all tasks requiring professional judgment including discussion of results and patient counseling. Technicians ended the encounter by documenting patient results within their respective electronic profiles. Health screening data was collected between February 2, 2020 – February 29, 2020 using the pharmacy software system and was reported via internal reporting mechanism.

Survey data collection occurred via an online platform (QuestionPro; Austin, TX).

The survey link was communicated to all Ohio and Tennessee pharmacists and pharmacy technicians using the weekly pharmacy newsletter and company email (where available) across a four-week period. The survey was designed by pharmacy leadership, staff, and University faculty, and inspired by the work of Weiner et al.^30^ that established acceptability, appropriateness and feasibility as valid and reliable measures of implementation outcomes. (Appendix A) The survey was reviewed internally, and content was revised based on feedback. All pharmacists and technicians in participating divisions had the opportunity to complete the survey. Participation was voluntary. Pharmacists and technicians responded to a series of previously validated questions to determine the acceptability, appropriateness, and feasibility of the services. Perceptions on additional concepts like job satisfaction were also collected.

To assess the primary objective, health screenings for 2019 and 2020 were compared using a paired *t*-test. Secondary objectives were analyzed using descriptive statistics. Results were analyzed using IBM SPSS Statistics version 25 (Armonk, NY).

Approval for this project was granted by the University of Tennessee Health Science Center (UTHSC) Institutional Review Board on February 26, 2020.

## 5 Results

Regarding the primary objective, participating pharmacies in Tennessee and Ohio performed 46% more health screenings in 2020 vs. 2019. (*p* < 0.0001).

The survey was distributed electronically to all participating pharmacists and technicians. 182 responses were completed after 20 dropouts (90.1% completion rate). The majority of respondents were female (68%, 123 of 180) and pharmacists (77%, 137 of 178). Table 1 provides a summary of responses regarding the acceptability, appropriateness, and feasibility of the service.

**Table 1:** Domains assessed in pharmacist/technician survey.

Acceptability	
Pharmacists	*n* = 144 (%)

I approve of technicians performing point-of-care testing (POCT).	
Strongly Disagree	7 (4.86%)
Disagree	7 (4.86%)
Slightly Disagree	2 (1.39%)
Slightly Agree	11 (7.64%)
Agree	54 (37.50%)
Strongly Agree	63 (43.75%)
I feel inspired to supervise technicians who are performing POCT activities.	
Strongly Disagree	9 (6.25%)
Disagree	9 (6.25%)
Slightly Disagree	6 (4.17%)
Slightly Agree	19 (13.19%)
Agree	49 (34.03%)
Strongly Agree	52 (36.11%)
I have no reservations delegating certain POCT activities to technicians.	
Strongly Disagree	7 (4.86%)
Disagree	12 (8.33%)
Slightly Disagree	4 (2.78%)
Slightly Agree	21 (14.58%)
Agree	42 (29.17%)
Strongly Agree	58 (40.28%)

Technicians	*n* = 41 (%)

I approve of technicians performing point-of-care testing (POCT).	
Strongly Disagree	1 (2.44%)
Disagree	1 (2.44%)
Slightly Disagree	2 (4.88%)
Slightly Agree	2 (4.88%)
Agree	14 (34.15%)
Strongly Agree	21 (51.22%)
I feel inspired to conduct POCT activities delegated to me by the pharmacist.	
Strongly Disagree	1 (2.44%)
Disagree	2 (4.88%)
Slightly Disagree	2 (4.88%)
Slightly Agree	2 (4.88%)
Agree	13 (31.71%)
Strongly Agree	21 (51.22%)
I have no reservations accepting responsibility for certain POCT activities.	
Strongly Disagree	1 (2.44%)
Disagree	1 (2.44%)
Slightly Disagree	3 (7.32%)
Slightly Agree	4 (9.76%)
Agree	13 (31.71%)
Strongly Agree	19 (46.34%)


Appropriateness	
All	*n* = 181 (%)
Pharmacy technicians performing POCT fulfills needed gaps in patient care.	
Strongly Disagree	7 (3.87%)
Disagree	6 (3.31%)
Slightly Disagree	10 (5.52%)
Slightly Agree	27 (14.92%)
Agree	64 (35.36%)
Strongly Agree	67 (37.02%)
Pharmacy technicians performing POCT seems suitable to improve pharmacy	workflow.
Strongly Disagree	8 (4.42%)
Disagree	11 (6.08%)
Slightly Disagree	9 (4.97%)
Slightly Agree	23 (12.71%)
Agree	49 (27.07%)
Strongly Agree	81 (44.75%)
Pharmacy technicians performing POCT seems applicable to my practice site.	
Strongly Disagree	6 (3.31%)
Disagree	9 (4.97%)
Slightly Disagree	7 (3.87%)
Slightly Agree	20 (11.05%)
Agree	61 (33.70%)
Strongly Agree	78 (43.09%)


Feasibility	
All	*n* = 182 (%)
Pharmacy technicians performing POCT activities can be implemented given current resources.	
Strongly Disagree	11 (6.04%)
Disagree	11 (6.04%)
Slightly Disagree	11 (6.04%)
Slightly Agree	24 (13.19%)
Agree	61 (33.52%)
Strongly Agree	64 (35.16%)
Pharmacy technicians performing POCT activities can be done without significant restructuring of pharmacy workflow.	
Strongly Disagree	17 (9.34%)
Disagree	15 (8.24%)
Slightly Disagree	25 (13.74%)
Slightly Agree	29 (15.93%)
Agree	57 (31.32%)
Strongly Agree	39 (21.43%)
Pharmacy technicians performing POCT activities is feasible given the preparedness of technicians to deliver the services.	
Strongly Disagree	11 (6.04%)
Disagree	14 (7.69%)
Slightly Disagree	8 (4.40%)
Slightly Agree	34 (18.68%)
Agree	58 (31.87%)
Strongly Agree	57 (31.32%)


Other Concepts	
All	*n* = 180 (%)
Pharmacy technicians performing POCT activities will enhance my job satisfaction.	
Strongly Disagree	13 (7.22%)
Disagree	11 (6.11%)
Slightly Disagree	12 (6.67%)
Slightly Agree	29 (16.11%)
Agree	62 (34.44%)
Strongly Agree	53 (29.44%)
Pharmacy technicians performing POCT activities will improve quality of work-life.	
Strongly Disagree	13 (7.22%)
Disagree	11 (6.11%)
Slightly Disagree	11 (6.11%)
Slightly Agree	30 (16.67%)
Agree	61 (33.89%)
Strongly Agree	54 (30.00%)
Pharmacy technicians performing POCT activities will broaden the scope of services offered by the pharmacy.	
Strongly Disagree	11 (6.11%)
Disagree	12 (6.67%)
Slightly Disagree	6 (3.33%)
Slightly Agree	32 (17.78%)
Agree	64 (35.56%)
Strongly Agree	55 (30.56%)

The majority of pharmacists agreed or strongly agreed that technicians performing POCT meets their approval (81%, 117 of 144). Technician-driven POCT meets the approval of 85% of CPhTs (34 of 41). Most pharmacy personnel believe that technician POCT fulfills needed gaps in patient care (72%, 131 of 181). Utilizing technicians for technical tasks is a suitable option to improve pharmacy workflow (72%, 130 of 181) and applicable at most practice sites (77%, 139 of 181).

Standardized training could be used to improve the feasibility of this service. 18% of pharmacy personnel disagree that this service is implementable given current resources (33 of 181). Over 30% believe that significant restructuring of pharmacy workflow is required (31%, 57 of 182). 64% of pharmacy personnel agree or strongly agree that this service can enhance job satisfaction and quality of work-life.

## 6 Discussion

Technician involvement in directly and indirectly supporting POCT services is a novel use of the pharmacy technician, though other studies have shown successful implementation of training for technicians in advanced roles.^1^^,^^2^^,^^15^ This study, then, adds to a growing body of evidence surrounding pharmacy technician support of expanding clinical services in the community pharmacy setting. Specifically, this study aimed to scale the innovative service shown to be accepted by patients and providers in the single-state U.S. pilot study by Hill et al.^9^ Our findings suggest what many others have found, which is that team-based task delegation to pharmacy support personnel may be a critical component for evolving the community pharmacy practice landscape as a healthcare service destination beyond procurement of drug therapy.

Gilson et al. found that redeployment of pharmacy support staff in a systems engineering model created greater patient engagement in the purchase of over-the-counter medications.^21^ Core to these re-deployments was that it made pharmacists and technicians more readily available for consultation. In the current study, an intervention not unlike theirs resulted in similar efficiencies in operations and an enhanced ability to deliver care. Other evidence suggests that strategic employment of technicians can result in an increase in the number of patients receiving vaccinations at the pharmacy.^22^ Hohmeier and Desselle described self-reported increases in development of new patient-centric services and an increase in the frequency of delivering existing services under the auspices of an Optimizing Care Model.^23^ Further research might consider whether and how interventions and/or re-engineering of jobs in the pharmacy can concurrently improve efficiency and measure objectively the delivery of various types of services concurrently, even while bearing mind that services must be prioritized and that it is logistically unfeasible for each pharmacy to make available every potential service to all of its patients, also notwithstanding the unnecessary duplication at a societal level.^24^ This too is a unique advantage of community pharmacies worldwide, as they intersect both healthcare delivery and retail services; the former emphasizing quality patient care that is convenient and the latter emphasizing the need to offer only products and services which represent the voice of their local customer.

There was widespread agreement among pharmacists and technicians that the deployment of technicians to assist in POCT was a positive change to workflow. Recent literature has been indicative of technicians' eagerness to embrace new roles.^25^ Similar sentiments have been expressed by pharmacists,^26^^,^^27^ and with regard to new technician roles and their advanced certification.^28^ The current study is among the first to examine pharmacists and technicians' attitudes concurrently following a specific intervention, or workflow redesign. It examined these attitudes specifically under the lens of implementation science theory, most notably the implementation outcomes framework^29^ and specifically those outcomes related to feasibility, appropriateness, and acceptability.^30^ This is an important lens under which to examine such attitudes, because a program or intervention with all three of these characteristics is more likely to be sustained in the long-term and is also more likely to achieve success in the midst of upscaling the project to a large number of locations.^31^^,^^32^

The finding that only a small percentage of pharmacists were hesitant to delegate responsibilities to technicians is of particular note. POCT and other emerging pharmacy services are more likely to succeed if pharmacists are willing and able to delegate various duties appropriately. Initial work has been done to evaluate pharmacists' effectiveness in delegating, with some promising results.^33^ The authors of that study point out that the ability to practice at the top of one's license hinges upon success in delegating administrative and supportive tasks. They point out, though, that some pharmacists still struggle with the idea that they must do everything themselves. This is perhaps why, that in spite of the clear economic advantages to job repurposing and the advances made in actuating patient-centric services, there still remain momentous gaps between what is currently taking place versus what is achievable.^34^

As such, more research is needed on effective delegation in pharmacy. That research might be assisted by or placed into context among the findings of Moya et al.^35^ These researchers performed a cluster analysis on technicians according to their current and desired involvement in various tasks. They identified groups of technicians already competent, those very willing embrace new roles and be trained, and those who did not and would prefer to stick with more traditional roles. Organizations and pharmacists in charge of hiring technicians might consider placing even greater emphasis on characteristics and behaviors like adaptability and flexibility in their willingness to have responsibilities delegated to them, in addition to further educating pharmacists on best practices when it comes to delegating to others.^36^

Adams describes the use of deregulation to expand scope of practice by pharmacy personnel, advocating for a small and particular list of activities precluded rather than a longer list of responsibilities that are permitted, which by default means that other activities are not allowed.^37^ In describing the evolution of expansion of roles of pharmacy support personnel, involvement, POCT is mentioned as among those activities as a logical component to expansion of pharmacist delegatory authority to technicians. This corroborates the findings of Smith and Rains;^38^ however, they found some barriers associated with implementation of a national training program for POCT. As such, more localized or more highly tailored training programs might be a better route, particularly if those programs have a solid foundation grounded in concepts such as the CFIR model and training in delegation, tailored in accordance with an organization's infrastructure, organizational mission, and culture. Of note, this was the approach used in the present study, where the pharmacy organization developed a training program in-house in consultation with academic researchers who were experts in POCT and advanced pharmacy technician roles.

POCT is also a key component of pharmacy's evolution, given an increased emphasis on patient activation and self-management, coinciding with pharmacy's increased focus on involvement in population health and wellness. Increasing the number of health screenings provided by pharmacies will significantly increase patient access to basic healthcare. Standardized training focuses on developing clear, supportive policies and training for this new, advanced role. With this in place, the service can be further scaled to accommodate expansion of technician roles in additional states as allowed by state regulation.

There were limitations in the present study. Study sites were only in the states of Tennessee and Ohio, thereby limiting the generalizability beyond the Southeast and Midwest U.S. These states were chosen based on favorable regulatory policies which allowed for advanced pharmacy technician role delegation to occur. It should be noted, however, that although the regional cultures in the Midwest and Southeast U.S. are distinctly different, that organizational climate was the same. Generalizability may be greater if the implementation took place in a multi-region pharmacy chain with an organizational climate which prioritizes clinical pharmacy services.

## Conclusion

This study provided supporting evidence that technician-supported POCT may positively impact the number of health screenings conducted in a community pharmacy setting. Standardization of training may allow for expansion of this service. Additionally, pharmacy personnel perceptions were overall positive. This study adds to a growing body of evidence that team-based task delegation of advanced pharmacy roles to pharmacy technicians is a key facilitator in increasing clinical service delivery in the community pharmacy setting.

## Disclosure

The authors declare no relevant conflicts of interest or financial relationships.

This research did not receive any specific grant from funding agencies in the public, commercial, or not-for-profit sectors.

## Previous presentations

The results of this study have been presented at the Virtual Research in Education and Practice Symposium, Chapel Hill, NC on June 22nd, 2020.

## Declaration of interests

The authors declare that they have no known competing financial interests or personal relationships that could have appeared to influence the work reported in this paper.

## Figures and Tables

**Table:** 

	Strongly disagree	Disagree	Slightly disagree	Slightly agree	Agree	Strongly agree
For Pharmacists						
1. I approve of technicians performing point-of-care testing (POCT).	①	②	③	④	➄	⑥
2. I feel inspired to supervise technicians who are performing POCT activities.	①	②	③	④	⑤	⑥
3. I have no reservations delegating certain POCT activities to technicians.	①	②	③	④	⑤	⑥
For Technicians						
1. I approve of technicians performing point-of-care testing (POCT).	①	②	③	④	⑤	⑥
2. I feel inspired to conduct POCT activities delegated to me by the pharmacist.	①	②	③	④	⑤	⑥
3. I have no reservations accepting responsibility for certain POCT activities.	①	②	③	④	⑤	⑥

**Table:** 

	Strongly disagree	Disagree	Slightly disagree	Slightly agree	Agree	Strongly agree
1. Pharmacy technicians performing POCT fulfills needed gaps in patient care.	①	②	③	④	⑤	⑥
2. Pharmacy technicians performing POCT seems suitable to improve pharmacy workflow.	①	②	③	④	⑤	⑥
3. Pharmacy technicians performing POCT seems applicable to my practice site.	①	②	③	④	⑤	⑥

**Table:** 

	Strongly disagree	Disagree	Slightly disagree	Slightly agree	Agree	Strongly agree
1. Pharmacy technicians performing POCT activities can be implemented given current resources.	①	②	③	④	⑤	⑥
2. Pharmacy technicians performing POCT activities can be done without significant restructuring of pharmacy workflow.	①	②	③	④	⑤	⑥
3. Pharmacy technicians performing POCT activities is feasible given the preparedness of technicians to deliver the service.	①	②	③	④	⑤	⑥

**Table:** 

	Strongly disagree	Disagree	Slightly disagree	Slightly agree	Agree	Strongly agree
1. Pharmacy technicians performing POCT activities will enhance my job satisfaction.	①	②	③	④	⑤	⑥
2. Pharmacy technicians performing POCT activities will improve quality of work-life.	①	②	③	④	⑤	⑥
3. Pharmacy technicians performing POCT activities will broaden the scope of services offered by the pharmacy.	①	②	③	④	⑤	⑥

## Appendix A

Pharmacist/Technician Survey

1. Please select your sex:

□ Female □ Male

2. Please provide your age: ______
3. Please select your title:
  □ Certified Pharmacy Technician □ RPh /PharmD
4. Please select your highest level of education:

□ High School Diploma □Associate's Degree □ Bachelor's Degree □ Doctorate Degree

5. Please select your total number of years in practice:

□ 0–5 □ 5–10 □ 10–15 □ 15–20 □ ≥ 20

**Acceptability questions**

**Appropriateness questions**

**Feasibility questions**

**Other concepts**

Please provide any additional comments to help us improve our services:
